# Tanreqing injection inhibits stemness and enhances sensitivity of non-small cell lung cancer models to gefitinib through ROS/STAT3 signaling pathway

**DOI:** 10.7150/jca.94438

**Published:** 2024-06-11

**Authors:** Zhenzhen Xiao, Lina Ding, Yaya Yu, Changju Ma, Chenjing Lei, Yihong Liu, Xuesong Chang, Yadong Chen, Yihan He, Yanjuan Zhu, Haibo Zhang

**Affiliations:** 1 Department of Oncology, The Second Affiliated Hospital of Guangzhou University of Chinese Medicine, Guangzhou, PR China.; 2 The Second Clinical College of Guangzhou University of Chinese Medicine, Guangzhou, PR China.; 3 Guangdong-Hong Kong-Macau Joint Lab on Chinese Medicine and Immune Disease Research, Guangzhou, PR China.; 4 Guangdong Provincial Key Laboratory of Clinical Research on Traditional Chinese Medicine Syndrome, Guangzhou, PR China.; 5 State Key Laboratory of Dampness Syndrome of Chinese Medicine, Guangzhou, PR China.

**Keywords:** gefitinib resistance, non-small-cell lung cancer, Tanreqing injection, stemness, ROS/STAT3 pathway

## Abstract

Resistance to epidermal growth factor receptor tyrosine kinase inhibitors (EGFR-TKIs) has emerged as a significant obstacle in managing patients with EGFR-mutant non-small-cell lung cancer (NSCLC), necessitating the exploration of novel therapeutic approaches. Tanreqing injection (TRQ) is a kind of Chinese patent medicine known for its heat-clearing and detoxifying properties. Studies have shown a correlation between tumor drug resistance and enrichment of cancer stem cells (CSCs). We aim to investigate the feasibility of TRQ enhancing sensitivity to gefitinib by targeting CSCs and reactive oxygen species (ROS). In our study, TRQ significantly inhibited cell proliferation in gefitinib-resistant non-small-cell lung cancer (NSCLC) models including 2D cell lines, 3D cell spheres, tumor-bearing animal and organoids. Compared with the gefitinib group alone, addition of TRQ elevated ROS levels, attenuated upregulation of the protein levels of sex-determining region Y-box 2 (SOX2) and aldehyde dehydrogenase 1 family member A1 (ALDH1A1) induced by gefitinib treatment, and inhibited the phosphorylation of signal transducer and activator of transcription 3 (STAT3). Scavenging ROS could restore tumor stemness, attenuate the inhibitory effect on the phosphorylation of STAT3, and promote cell proliferation. These results suggested that TRQ could enhance sensitivity of NSCLC models to gefitinib, providing a new combined treatment strategy.

## Introduction

Lung cancer is the leading cause of cancer-related death among malignant tumors in the world and remains a serious threat to human health 1. Epidermal growth factor receptor (EGFR) mutations account for approximately 10% to 40% of non-small cell lung cancer (NSCLC) and confer sensitivity to the EGFR tyrosine kinase inhibitors (EGFR-TKIs) 2. Unfortunately, a significant proportion of patients with EGFR-mutant NSCLC receiving EGFR-TKIs treatment ultimately develop disease progression, EGFR-TKIs resistance has been an obstacle in clinical management. In addition to a primary drug resistance rate of approximately 20%, patients who initially respond well to EGFR-TKIs may develop acquired resistance within 9.8 to 17.8 months of treatment 3. Although EGFR T790M-mediated resistance has been overcome by the third generation of EGFR-TKIs such as osimertinib, there are still many EGFR-independent resistance mechanisms including genetic and other signaling aberrations 4. Current targeted therapeutic strategies for EGFR-TKIs resistance are limited. Therefore, it is of quite necessary to seek new and effective therapeutic methods.

Traditional Chinese Medicine (TCM) offers unique advantages of enhancing efficacy and reducing toxicity in the clinical treatment of malignant tumors. Our team has focused on determining the optimal therapeutic principles for combining TCM with EGFR-TKIs to achieve the best outcomes. Previous studies 5-6 have suggested that incorporating heat-clearing TCM is essential when combining TCM therapies with EGFR-TKIs. Tanreqing injection (TRQ), a kind of Chinese patent medicine, is consist of wild honeysuckle flower, baical skullcap root, forsythiae fructus, goral horn, and bear gall powder. TRQ has the effects of heat-clearing, removing phlegm and detoxifying, approved clinically for the treatment of respiratory-related diseases in China. Multiple meta-analyses 7-8 have demonstrated TRQ's effectiveness that TRQ had a significant effect in the combination treatment of acute exacerbation of chronic obstructive pulmonary disease and cough caused by acute trachea-bronchitis, there is limited research on TRQ as a combination therapy for lung cancer.

Cancer stem cells (CSCs) have unlimited self-renewal and differentiation ability, and depend on their own metabolism for survival, which is an important cause of EGFR-TKIs resistance 9. Signal transducer and activator of transcription 3 (STAT3) is a signal transducer and activator of transcription. The continuously activated STAT3 signaling pathway is not only closely related to tumor growth, but also promotes the stemness of tumor cells, which leads to drug resistance 10. Reactive oxygen species (ROS) is the main molecules produced during oxidative stress. The ROS level in drug-resistant tumor cells is frequently low, and an increased ROS level can effectively decrease chemoresistance and encourage the death of chemoresistant cells 11. Research findings indicate that the inhibition of the ROS/STAT3 signaling pathway can impede the growth of cancer stem cells 12. However, whether TRQ can exert anti-tumor effect through this mechanism is still unclear.

According to our earlier findings 13, 16.67% of the EGFR co-mutated genes most strongly linked to resistance were PIK3CA mutations. Admittedly, acquisition of T790M mutation is a significant cause to drive resistance 14. Therefore, in this study, constructed PC-9-PIK3CA-mutation (PC-9-PIK3CA-M) cell lines (EGFR ex19del /PIK3CA) 15, H1975 cell lines (EGFR L858R/T790M) and H1650 cell lines (EGFR ex19del/ PTEN del) 16 were selected to observe the anti-tumor effect of TRQ combined with gefitinib and explore its potential mechanism.

## Materials and Methods

### Reagents and cell lines

Gefitinib (S1025) and N-acetyl-l-cysteine (S1623) were obtained from Selleck Co., Ltd (Shanghai, China). TRQ was purchased from Kai-Bao Pharmaceutical Co., Ltd (Shanghai, China, Z20030054).

Human PC-9 cells (EGFR ex19del) were obtained from the Cell Line Bank at the Laboratory Animal Center of Sun Yat-sen University (Guangzhou, China), Human H1975 cells and H1650 cells were purchased from National Collection of Authenticated Cell Cultures (Shanghai, China). The complete culture medium consisted of RPMI-1640 medium containing 10% fetal bovine serum (FBS) and 100 U/mL penicillin and 100 μg/mL streptomycin. The construction of PC-9-PIK3CA-M cell lines have been described and confirmed previously 15 and were cultured in complete culture medium supplemented with 2.5 μg/mL puromycin (Mpbio, CA, USA, 219453925). Human bronchial epithelial cells (16-HBE) were purchased from National Collection of Authenticated Cell Cultures (Shanghai, China) and maintained in glucose Dulbecco's modified Eagle's medium (DMEM) supplemented with 10% FBS and 100 U/mL penicillin and 100 μg/mL streptomycin. Cell lines at passage 3 were used for subsequent experiments and were cultured at 37°C, 5% CO_2_.

### 3D lung cancer cell spheres culture

The DMEM/F12 medium was added with 25 ng/mL epidermal growth factor (EGF, AF-100-15, PeproTech, NJ, USA), 10 ng/mL basic fibroblast growth factor (bFGF, AF-100-18C, PeproTech), 5 mM Y-27632(129830382, AbMole, Houston, USA) and 2% B27 Supplement (17504044, Gibco, CA, USA). Cells were suspended in matrigel (356231, Corning, NYC, USA) at 4°C and seeded into cell well plates. Then cell well plates were put upside down for 30 min at 37℃ until the matrigel solidified. Human PC-9-PIK3CA-M cells were maintained in DMEM/F12 medium with 2.5 μg/mL puromycin for 5 days, human H1975 cells and H1650 cells were cultured in DMEM/F12 medium for 7-9 days.

### Chromatographic assay

Filter 1 mL of the original TRQ injection solution through a 0.22 μm microporous membrane. Use the filtrate to perform the injection test. Baicalin, ursodeoxycholic acid, and chenodeoxycholic acid were utilized as control chemicals. The chromatography column was 250 mm × 4.6 mm, 5 μm. Phase A's mobile phase was 0.1% formic acid-water, whereas Phase B's was acetonitrile. The flow rate was set at 1.0 mL. The flow rate was 1.0 mL/min. The column temperature was 25℃. The elution protocol was as follows: from 0 to 25 min, B phase: 8% to 8%; from 25 to 30 min, B phase: 8% to 19%; and from 30 to 50 min, B phase: 19% to 19%.

### Cell viability assay

Cell lines (3 × 10^3^ cells) were seeded in 96-well plates and treated with drugs alone or in combination for 48 h. 10 μL Cell Counting Kit-8(CCK-8, G9682, Yeasen, Shanghai, China) reagent was added to the wells for 30 min-1 h at 37°C with 5% CO_2_. Afterward, a microplate reader was used to determine the absorbance at 450 nm.

Cell spheres (5 × 10^3^ cells) were cultured in 384-well plates with 100 μL medium. After treated with different drugs for 48 h, the proliferation of cell spheres was detected by CellTiter-Glo® 3D Cell Viability Assay (G968A, Promega, Madison, USA).

### Apoptosis assay

Cell lines (1.5 × 10^5^ cells) were seeded in 6-well plates and cell spheres (3 × 10^4^ cells) were cultured in 24-well plates. Then, cells were treated with different drugs for 48 h. After harvested, washed, cell lines were resuspended with phosphate-buffered saline (PBS), cell spheres were collected by cell recovery solution (354253, Corning). Apoptotic cells were stained according to the manufacturer's protocol of Annexin V-FITC/PI Apoptosis Detection Kit (556547, BD, NJ, USA). Then a flow cytometer was used to analyze cell apoptosis.

### Quantitative Real-Time PCR (qPCR)

After treatments, cells and recovered cell spheres were resuspended in trizol reagent. Total RNA was extracted, cDNA template was synthesized following the instructions of HiScript III RT SuperMix for qPCR (+gDNA wiper) (R323, Vazyme, Nanjing, China). The amplification was carried out according to the instructions of ChamQ SYBR qPCR Master Mix (Q711, Vazyme) by Quantitative Real-Time PCR System. Protocol was as follows: 95℃ for 30 s, 40 cycles of 95℃ for 10 s, 60℃ for 30 s. 2^-ΔΔCt^ method was used to analyze the relative expression of mRNA. The sequences of each primer were shown as follows: SOX2 Forward 5'-TACTGGCGAACCATCTCTGTG-3', Reverse 5'-ACCAACGGTGTCAACCTGC-3'; ALDH1A1 Forward: 5'-CTGCTGGCGACAATGGAGT-3', Reverse 5'-CCATCAATTGGTATTGTACGGC-3'.

### Western blotting

Total cell protein was extracted from cell lysis buffer containing protease and phosphatase inhibitors. After concentration of proteins was determined, 25 μg protein samples were separated on 10% gels, transferred onto the 0.45 μm PVDF membranes, blocked with 5% skim milk powder for 90 min, then incubated with rabbit anti-human SOX2(1:1000, 23064), ALDH1A1(1:1000, 36671), p-STAT3(1:2000, 9145), STAT3(1:2000, 4904), GAPDH (1:10000, 2118), *β*-Actin (1:10000, 3700) antibodies (CST, Boston, USA) overnight at 4°C. Then, the membranes were washed three times and incubated with secondary antibodies (1:10000, BA1054, Boster, Wuhan, China) for 1 h at room temperature. After washed three times, membranes were scanned by an imaging system. ImageLab software was used to quantify the intensity of the immune-reactive bands.

### Cell redox ratio detection

Endogenous fluorophores NAD(P)H and FAD of cell spheres were detected by femtosecond multiphoton microscopy (MPM) without sectioning, staining, or labeling 17. Cell redox ratio (FAD/[FAD+NADH]) was calculated. It was reported that the optical redox ratio was highly consistent with the redox ratio based on mass spectrometry 18-20. Cell spheres were cultured in *μ*-Slide 8 well at a density of 5 ×10^4^ cells/well, images were acquired by femtosecond label-free imaging (FLI) microscopy system (Femtosecond research center, Guangzhou, China).

### ROS detection *in vitro*

Intracellular total ROS was quantified following the instructions of by Cell Meter™ Fluorimetric Intracellular Total ROS Activity Assay Kit (22902, AAT Bioquest, CA, USA). Mitochondrial superoxide was detected by Cell Meter™ Fluorimetric Mitochondrial Superoxide Activity Assay Kit (16060, AAT Bioquest). After incubated for 1 h with corresponding working solution, cells were observed by a confocal laser scanning microscope and inverted fluorescence microscope.

### Xenografted tumor model

Nu/Nu mice (20 ± 2 g) were obtained from Guangdong Vital River Experimental Animal Co. Ltd (Guangdong, China), and maintained in the Animal Center of Guangdong Provincial Hospital of Chinese Medicine (Guangdong, China, SYXK 2018-0094). All experimental procedures were approved by the Animal Care and Use Committee of Guangdong Provincial Hospital of Chinese Medicine (Ethics Approval Number 2022019), and conducted according to the Guide for Care and Use of Laboratory Animals of the National Institute of Health.

PC-9-PIK3CA-M cell (2 × 10^6^ cells) were resuspended with PBS and injected subcutaneously into the right forelimb of the nude mice. When tumors became palpable (~50 mm^3^), mice were randomly divided into four groups: control group (*n* = 5), gefitinib treatment group (2.5 mg/kg) (*n* = 5), TRQ treatment group (10 mL/kg) (*n* = 5), gefitinib plus TRQ treatment group (*n* = 5). Duration of administration for one month, body weight and tumor size of mice were measured once 3-4 days. All mice were sacrificed by anesthetics for subsequent experiments.

### ROS detection* in vivo*

The tumor tissues were washed, cut into smaller pieces and transferred into digestion buffer. It was then incubated for 30-60 min at 37°C while shaking gently. The cells were filtered over a 70 µm cell strainer, washed, and the remaining cells were resuspended in the corresponding amount of working solution according to instructions of Cell Meter™ Fluorimetric Intracellular Total ROS Activity Assay Kit and Cell Meter™ Fluorimetric Mitochondrial Superoxide Activity Assay Kit. The levels of total ROS and mitochondrial superoxide were monitored by a flow cytometer.

### Organoid culture

Human samples of lung cancer were derived from puncture tissue or pleural fluid specimens with patients' informed consent. Research protocol was approved by the Ethics Committee of Guangdong Provincial Hospital of Chinese Medicine (Guangdong, China, BF2020-017). The entire experimental protocol was conducted following the institutional guidelines. Samples were confirmed as NSCLC on the basis of histopathological assessment.

The Organoid culture medium was composed of advanced DMEM/F12 supplemented with 1% penicillin/streptomycin, 10 ng/mL bFGF, 50 ng/mL EGF, 10 μM Y-27632, 10 mM Nicotinamide (AbMole, Houston, USA, M4896), and 1 × HEPES, 1 × Glutamax, 1 × N2, 2% B27 (all from Gibco), 1.25 mM N-Acetylcysteine, 1 μM SB202190 (S7067) (all from Sigma, MO, USA), 250 ng/mL R-spondin-1 (11083), 100 ng /mL Noggin (10267), 10 ng/mL FGF10 (10573) (all from Sino, Beijing, China), A83-01 (Absin, Shanghai, China).

### Statistical analysis

Data were shown as means ± standard deviation (SD). Statistical analyses were performed using GraphPad Prism 8. Unpaired Student's t-test was used to compare two groups, comparison between multiple groups was assessed by one-way ANOVA. *P-* value < 0.05 was considered as statistically significant.

## Results

### TRQ combined with gefitinib inhibited cell proliferation and promoted apoptosis in NSCLC 2D cell lines

**Figure 1:**
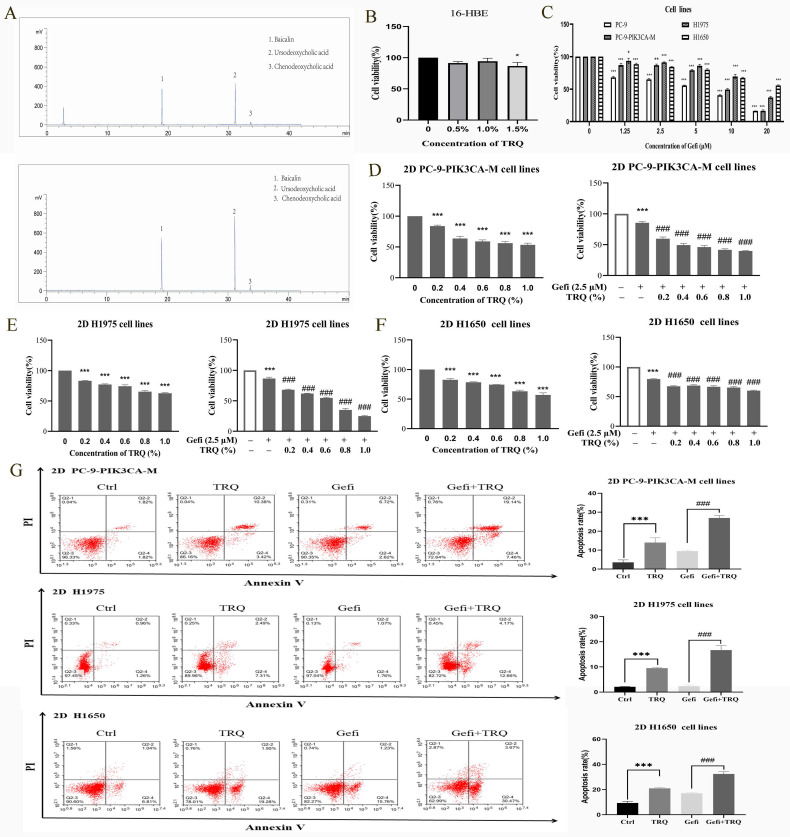
** TRQ combined with gefitinib inhibited cell proliferation and promoted apoptosis in NSCLC 2D cell lines.** (A) The Chromatographic assay of TRQ and major components including Baicalin, Ursodeoxycholic acid and Chenodeoxycholic acid. (B, C) After cell lines were treated with TRQ or gefitinib for 48 h, CCK-8 assay was performed to detect the cell viability. (D-F) The effect of TRQ alone or in combination with gefitinib on proliferation of lung cancer cells. (G) Cell apoptosis rate was detected after treated with gefitinib (2.5 μM), TRQ (0.6%) alone or together in lung cancer cells. Data were shown as means ± SD of three independent experiments. *P < 0.05 versus the control group, #*P* < 0.05 versus the gefitinib alone group, **P or ^##^*P* < 0.01, ***P or ^###^*P* < 0.001. TRQ, Tanreqing injection; Gefi, gefitinib; PC-9-PIK3CA-M, PC-9-PIK3CA-mutation.

The chromatographic analysis data were presented as listed in figure 1A. We first determined that the safe concentration of TRQ in normal human bronchial epithelial cells 16-HBE was less than 1% (Figure 1B), and then screened the appropriate concentration of gefitinib (Figure 1C). Gefitinib (2.5 μM) was used to detect the combined efficacy of gefitinib and TRQ *in vitro*. Compared with the control group, TRQ significantly inhibited the proliferation of lung cancer cells. Moreover, TRQ combined with gefitinib could enhance the inhibitory effect of gefitinib on the viability of lung cancer cells (Figure 1D-F).

According to previous studies, lung cancer cells were exposed to TRQ (0.6%) and gefitinib (2.5 μM). The results illustrated in Figure 1G demonstrate that TRQ notably enhanced the apoptosis of lung cancer cells when compared to the control group. Furthermore, the apoptosis rate was significantly higher in the group treated with both TRQ and gefitinib compared to the group treated with gefitinib.

### TRQ combined with gefitinib inhibited cell proliferation and promoted apoptosis in NSCLC 3D cell spheres

**Figure 2:**
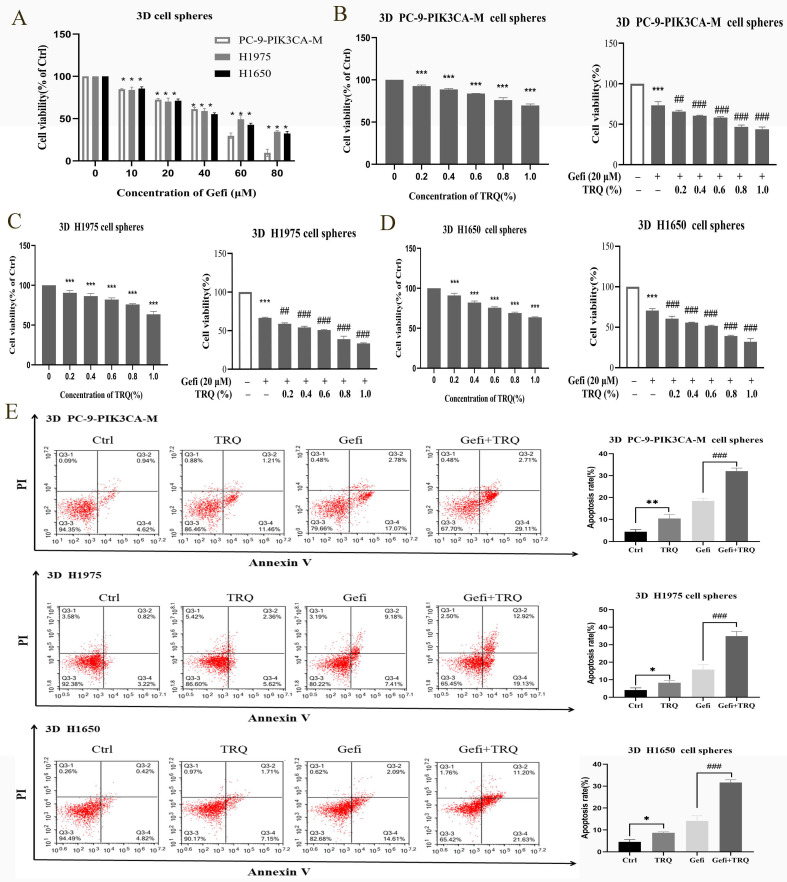
**TRQ combined with gefitinib inhibited cell proliferation and promoted apoptosis in NSCLC 3D cell spheres.** (A) Cell proliferation was detected after treated with gefitinib for 48 h. (B-D) The effect of TRQ alone or in combination with gefitinib on proliferation of 3D lung cancer cell spheres. (E) Cell apoptosis rate was detected after treated with Gefi (20 μM), TRQ (0.8%) alone or together in 3D cell spheres. Data were shown as means ± SD of three independent experiments. ^#^*P* < 0.05 versus the gefitinib alone group, ^###^*P* < 0.001. Ctrl, the control group; TRQ, Tanreqing injection; Gefi, gefitinib; PC-9-PIK3CA-M, PC-9-PIK3CA-mutation.

According to proliferation results of NSCLC 3D cell spheres treated with gefitinib (Figure 2A), gefitinib (20 μM) was used in combination with various concentrations of TRQ (0.2%, 0.4%, 0.6%, 0.8%, 1.0%) to detect the cooperative effect in 3D cell spheres. As depicted in Figure 2B-D, TRQ alone or in combination with gefitinib had apparent inhibitory effect on the viability of NSCLC 3D cell spheres.

In line with Figure 2E, TRQ demonstrated a significant enhancement in promoting apoptosis of 3D cell spheres compared to the control group. Additionally, the apoptosis rate was notably increased in NSCLC 3D cell spheres when TRQ was combined with gefitinib, as opposed to gefitinib alone.

### Combination treatment with gefitinib and TRQ significantly increased cell redox ratio and ROS levels in NSCLC models

**Figure 3:**
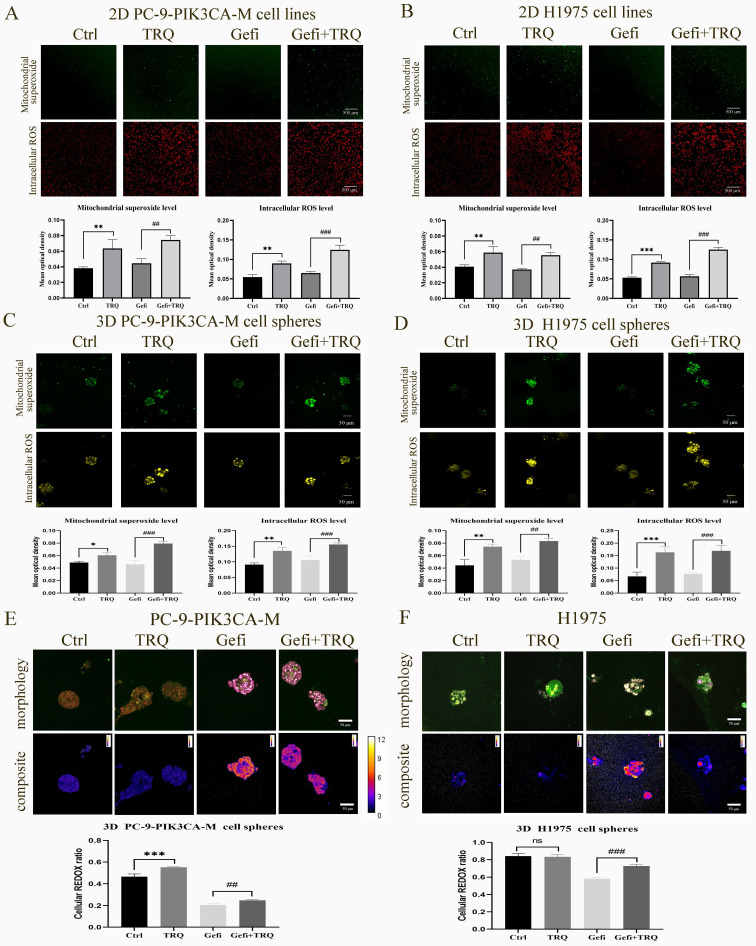
**Combination treatment with gefitinib and TRQ significantly increased ROS levels and cell redox ratio in NSCLC models.** (A, B) Representative confocal image of mitochondrial superoxide (green, scale bars, 500 μm) and intracellular ROS (red, scale bars, 500 μm) in 2D cell lines after treated with gefitinib (2.5 μM), TRQ (0.6%) alone or together. (C, D) Representative confocal image of mitochondrial superoxide (green, scale bars, 50 μm) and intracellular ROS (yellow, scale bars, 50 μm) in 3D cell spheres after treated with gefitinib (20 μM), TRQ (0.8%) alone or together. (E, F) Representative morphology image and composite image in cell spheres after treated with gefitinib (20 μM), TRQ (0.8%) alone or together (scale bars, 50 μm). The higher the value was, the lower the cell redox ratio (FAD/[NADH+FAD]) was. The results are represented as mean ± SD. ^*^P < 0.05 versus the control group, ^#^*P* < 0.05 versus the gefitinib alone group,^ **^P or ^##^*P* < 0.01, ^***^P or ^###^*P* < 0.001. Ctrl, the control group; TRQ, Tanreqing injection; Gefi, gefitinib; PC-9-PIK3CA-M, PC-9-PIK3CA-mutation.

To determine whether TRQ enhanced the anti-tumor of gefitinib by inducing ROS, we examined the ROS levels in 2D PC-9-PIK3CA-M and H1975 cell lines and 3D PC-9-PIK3CA-M and H1975 cell spheres exposed to gefitinib or TRQ alone, or their combination. As shown in Figure 3A-D, compared to the control group, TRQ significantly increased the intracellular ROS levels and mitochondrial superoxide levels. And compared to the gefitinib alone group, TRQ combined with gefitinib further elevated the ROS levels.

Redox ratio in PC-9-PIK3CA-M and H1975 cell spheres was detected by femtosecond laser unmarked imaging technology, and morphology image and composite image were shown in Figure 3E, F. In the composite image, the higher the ratio of NADH/FAD was, the lower the cell redox ratio (FAD/[NADH+FAD]) became, the redder the color was, whereas the bluer the color was. We found that the combination with gefitinib and TRQ could significantly increase cell redox ratio.

### TRQ reversed gefitinib-induced upregulation of stem cell markers by regulating ROS

**Figure 4:**
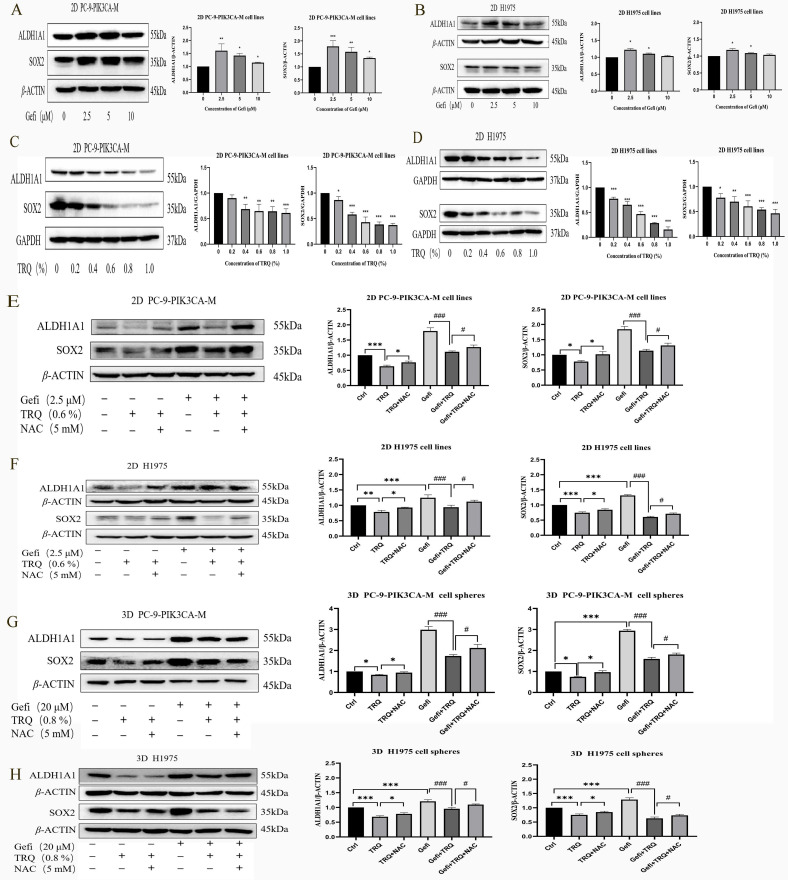
** TRQ inhibited certain stem cell markers related to resistance to gefitinib by modulating ROS levels.** (A, B) Western blotting assay was used to detect the protein levels of SOX2 and ALDH1A1 in lung cancer cell lines after gefitinib treatment. (C, D) Western blotting assay was used to detect the protein levels of SOX2 and ALDH1A1 in lung cancer cell lines after TRQ treatment. (E, F) Western blotting assay was used to detect the protein levels of SOX2 and ALDH1A1 after treated with gefitinib, TRQ, NAC alone or together in 2D cell lines. (G, H) Western blotting assay was used to detect the protein levels of SOX2 and ALDH1A1 after treated with gefitinib, TRQ, NAC alone or together in 3D cell spheres. Data were shown as means ± SD of three independent experiments.^ *^P or ^#^*P* < 0.05, ^**^P or ^##^*P* < 0.01,^ ***^P or ^###^*P* < 0.001. Ctrl, the control group; TRQ, Tanreqing injection; Gefi, gefitinib; NAC, N-acetyl-l-cysteine; PC-9-PIK3CA-M, PC-9-PIK3CA-mutation; SOX2, sex determining region Y-box 2; ALDH1A1, aldehyde dehydrogenase family 1 memberA1.

Targeting cancer stem cells is one of the important strategies to reverse EGFR-TKIs resistance. As shown in Figure 4A, B, protein levels of CSCs markers SOX2 and ALDH1A1 were upregulated after gefitinib treatment. To understand how TRQ overcame the resistance to gefitinib, stem cell markers were detected following treatment with TRQ alone or in combination with gefitinib. The results indicated that TRQ significantly down-regulated the protein levels of SOX2 and ALDH1A1 in PC-9-PIK3CA-M cells and H1975 cells compared with the control group (Figure 4C, D). Further investigation into the combined effect of TRQ and gefitinib on stem cell markers revealed that TRQ reversed the upregulation of SOX2 and ALDH1A1 induced by gefitinib, with this reversal was attenuated by ROS inhibitor NAC (Figure 4E, F). Similar results were observed in 3D cell spheres, where TRQ demonstrated synergy with gefitinib in inhibiting the protein levels of SOX2 and ALDH1A1 induced by gefitinib. Additionally, after further treatment with NAC, the down-regulated trend was also restored. (Figure 4G, H).

### TRQ inhibited cell proliferation by down-regulating the ROS-mediated STAT3 signaling pathway

**Figure 5:**
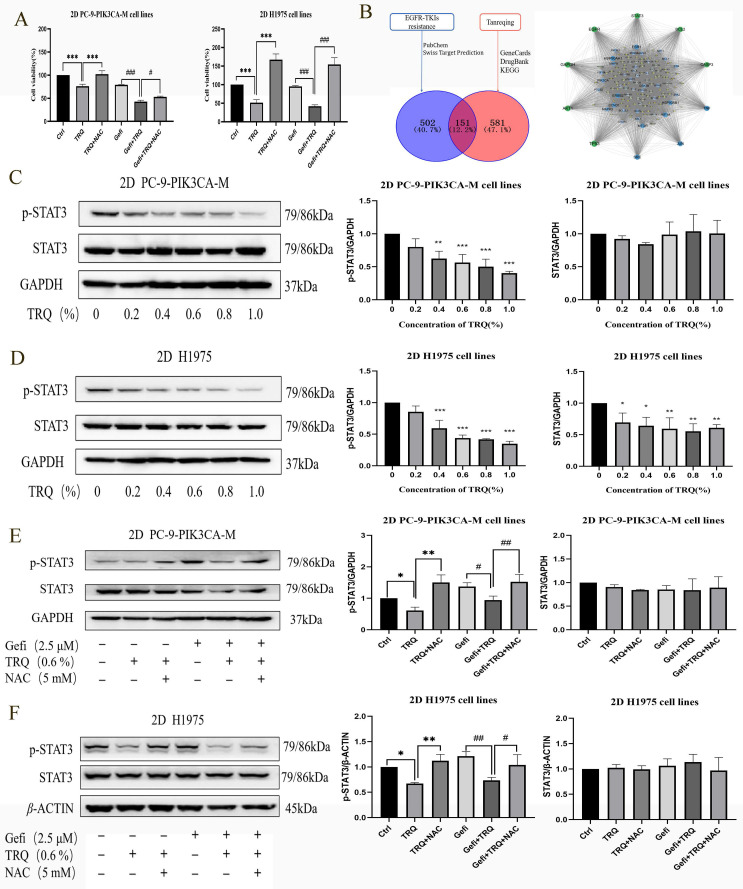
** TRQ inhibited cell proliferation by down-regulating the ROS-mediated STAT3 signaling pathway** (A) CCK-8 assay was performed to detect the cell viability after cell lines were treated with TRQ, gefitinib, NAC alone or together for 48 h. (B) Database and PPI network were used to screen the possible targets between TRQ and EGFR-TKIs resistance. (C, D) Western blotting assay was used to detect the STAT3 protein and its phosphorylation level in lung cancer cell lines after TRQ treatment. (E, F) Western blotting was used to detect the STAT3 protein and its phosphorylation level after treated with gefitinib, TRQ, NAC alone or together in 2D cell lines. Data were shown as means ± SD of three independent experiments.^ *^P or ^#^*P* < 0.05, ^**^P or ^##^*P* < 0.01,^ ***^P or ^###^*P* < 0.001. Ctrl, the control group; TRQ, Tanreqing injection; Gefi, gefitinib; NAC, N-acetyl-l-cysteine; PC-9-PIK3CA-M, PC-9-PIK3CA-mutation.

To investigate whether TRQ inhibited cell proliferation through ROS, NAC intervention was employed. The findings revealed that the introduction of NAC reversed the suppression of cell survival rates caused by TRQ alone or in combination with gefitinib (Figure 5A). Furthermore, targets related to EGFR-TKIs resistance and the main components of TRQ were screened, and a protein-protein interaction (PPI) network was constructed using Cytoscape and STRING platforms. The network analysis revealed that STAT3 could be a potential target linking TRQ and EGFR-TKIs resistance (Figure 5B). In gefitinib-resistant cell lines, TRQ treatment led to a significant decrease in the phosphorylation of STAT3 protein at various concentrations compared to the control group (Figure 5C, D). Moreover, the combination of TRQ and gefitinib resulted in a notable reduction in STAT3 phosphorylation compared to gefitinib alone (Figure 5E, F). Interestingly, NAC intervention reversed the inhibition of STAT3 phosphorylation observed with TRQ treatment.

### TRQ enhanced the antitumor activity of gefitinib via inhibiting CSCs and improving ROS levels in NSCLC xenograft model

**Figure 6:**
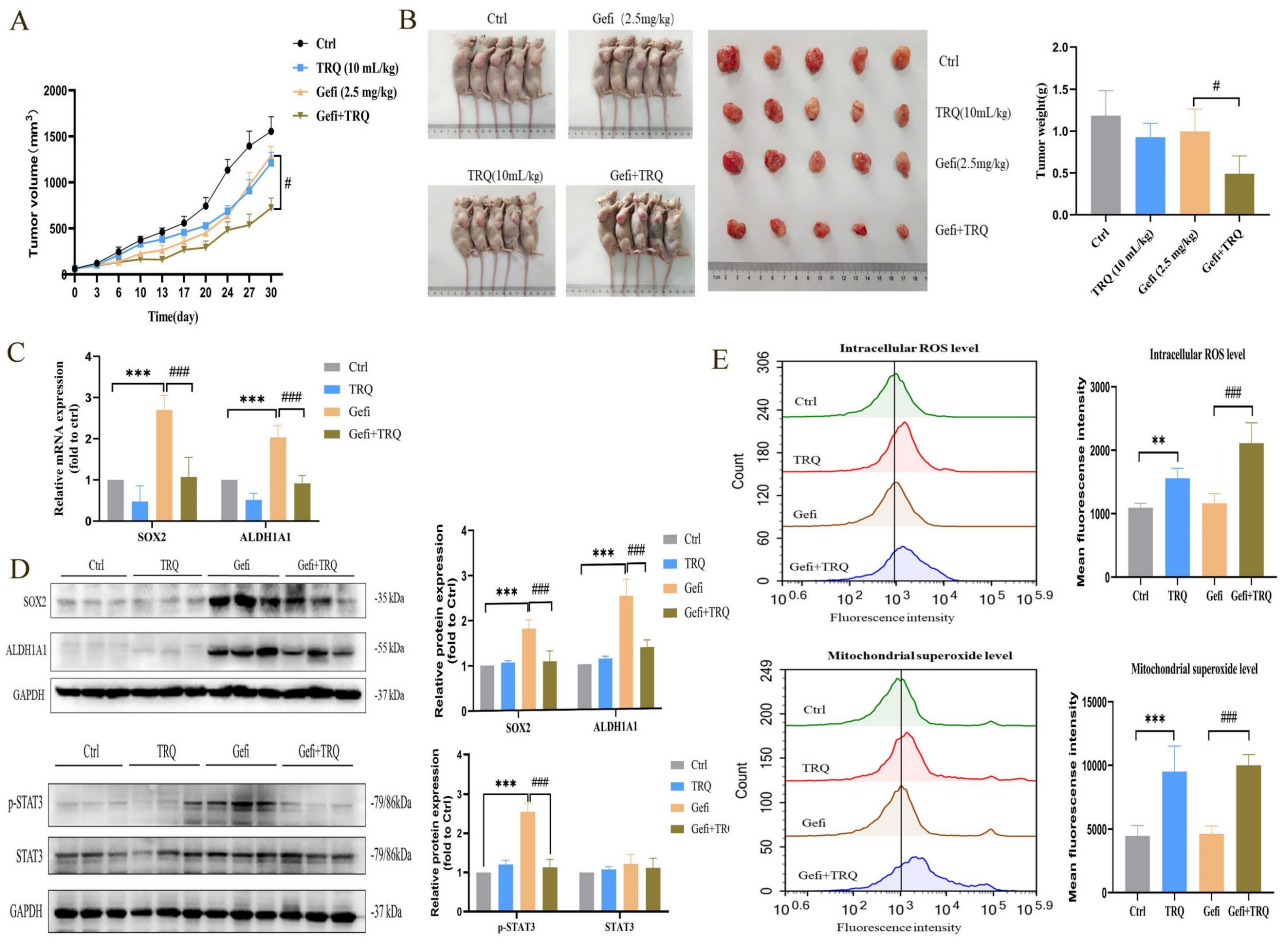
** Combination treatment with gefitinib and TRQ inhibits the tumor growth in PC-9-PIK3CA-M xenograft model.** (A) Evolution of tumor volumes of PC-9-PIK3CA-M xenograft model during treatment. (B) Representative PC-9-PIK3CA-M xenograft mice and tumors surgically removed. (C, D) The mRNA expression of SOX2, ALDH1A1 was assayed by PCR and protein levels of SOX2, ALDH1A1, p-STAT3 in tumors was detected by western blotting. (E) The intracellular ROS levels and mitochondrial superoxide levels in tumors was assayed by flow cytometry. Data were shown as means ± SD (*n* = 5).^ *^P < 0.05 versus the control group, ^#^*P* < 0.05 versus the gefitinib alone group,^ **^P or ^##^*P* < 0.01, ^***^P or ^###^*P* < 0.001. Ctrl, the control group; TRQ, Tanreqing injection; Gefi, gefitinib; SOX2, sex determining region Y-box 2; ALDH1A1, aldehyde dehydrogenase family 1 member A1.

Xenograft models were utilized to investigate the potential of TRQ to enhance the efficacy of gefitinib in treating tumors. PC-9-PIK3CA-M cells were inoculated subcutaneously into Nu/Nu nude mice. The mice were then treated with gefitinib, TRQ, or the combination of gefitinib/TRQ, for almost 30 days. Our results indicated that the combination of TRQ and gefitinib exhibited a stronger suppression of tumor growth compared to treatment with gefitinib alone (Figure 6A, B). We further investigated the effects of TRQ, gefitinib, and their combination on stem cell markers and STAT3 phosphorylation. The change trend of stemness was consistent with cell lines and cell spheres, moreover, the combination of TRQ and gefitinib resulted in a notable reduction in STAT3 phosphorylation compared to gefitinib alone (Figure 6C, D). In addition, to further explore the effects of combination treatment with gefitinib and TRQ on ROS levels, ROS levels in each group were detected by flow cytometry. As illustrated in Figure 6E, TRQ notably increased intracellular ROS levels and mitochondrial superoxide levels compared to the control group. This effect was more pronounced when TRQ was combined with gefitinib than when gefitinib was administered alone.

### Establishment of patient-derived organoids to further validate antitumor efficacy of combination treatment with gefitinib and TRQ

**Figure 7:**
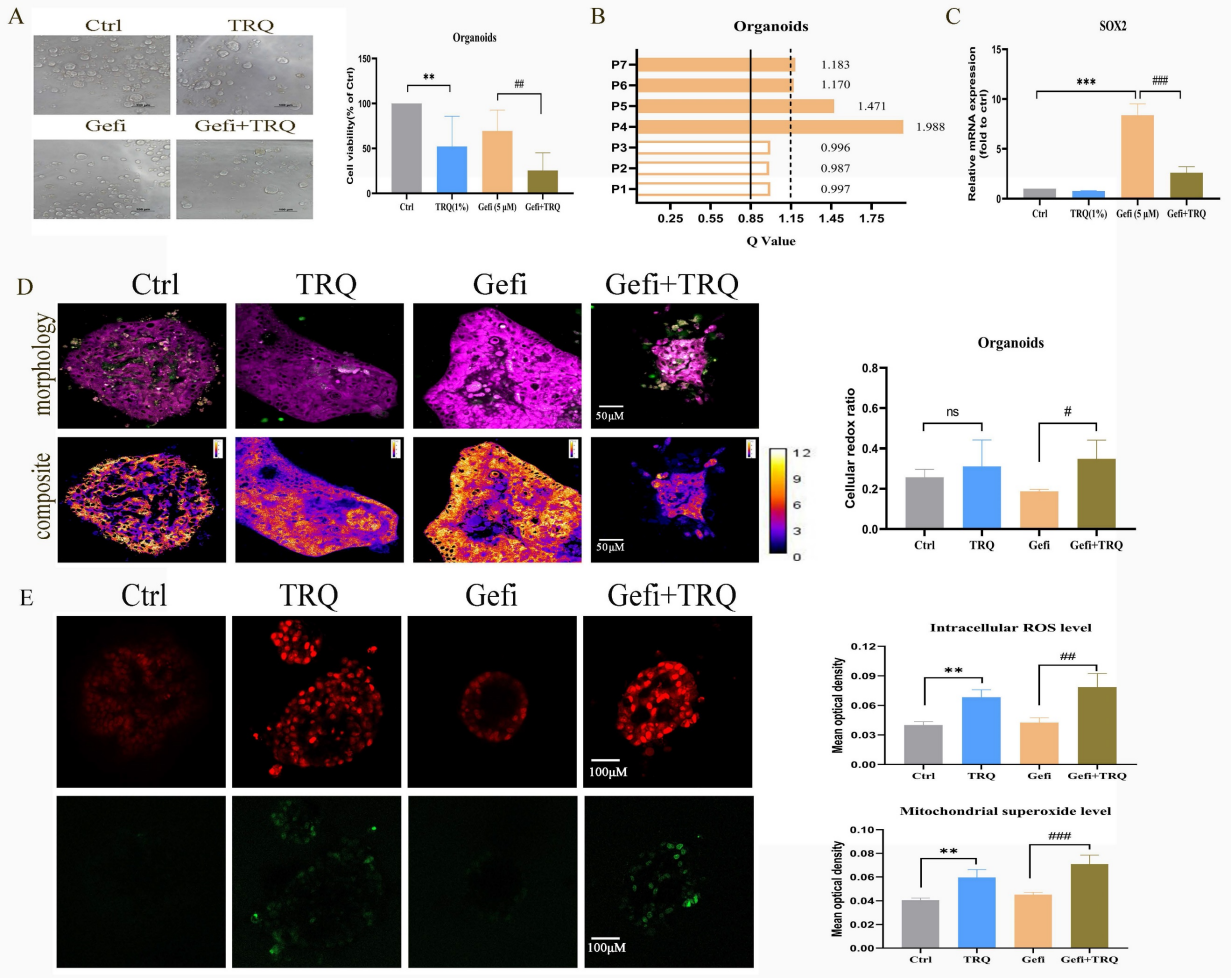
** Combination treatment with gefitinib and TRQ inhibits the organoids growth.** (A) Seven organoids were treated with TRQ or gefitinib or their combination for 72 h, CellTiter-Glo® 3D Cell Viability Assay was performed to detect the cell viability (*n* = 7). (B) The Q value method was used to assess the combined effect in seven organoids. The Q values were calculated from the average inhibition rate of each group. (C) The mRNA expression of SOX2 in organoids was assayed by PCR (*n* = 3). (D) Representative morphology image and composite image in organoids (scale bars, 50 μm) (*n* = 4). (E) Representative image of intracellular ROS (red, scale bars, 100 μm) and mitochondrial superoxide (green, scale bars, 100 μm) in organoids (*n* = 3). The results were represented as mean ± SD. ^*^P < 0.05 versus the control group, ^#^*P* < 0.05 versus the gefitinib alone group,^ **^P or ^##^*P* < 0.01, ^***^P or ^###^*P* < 0.001. Ctrl, the control group; TRQ, Tanreqing injection; Gefi, gefitinib; SOX2, sex determining region Y-box 2.

In order to further verify the clinical effect of TRQ combined with gefitinib, we selected the preclinical model-organoids for follow-up experiments. Seven organoids derived from patients with advanced NSCLC resistant to EGFR-TKIs were analyzed, with clinical features detailed in Table 1. Following successful construction of the organoid models, treatment with gefitinib (5 μM) alone, TRQ (1%) alone, or a combination of both was administered to assess cell viability. The Q value method was utilized to calculate the combined effect. Results indicated that TRQ significantly inhibited organoid proliferation compared to the control group. Furthermore, the combination of TRQ with gefitinib enhanced the inhibitory effect of gefitinib on organoid viability (Figure 7A). Subsequent analysis revealed that four out of seven organoids exhibited synergistic effects (Q > 1.15), while the remaining three showed only additive effects (0.85 < Q ≤ 1.15) (Figure 7B). Additionally, gefitinib treatment led to a significant up-regulation of SOX2 mRNA expression, which was partially reversed by TRQ addition (Figure 7C). Moreover, the combination of TRQ and gefitinib resulted in increased cell redox ratio, intracellular ROS levels, and mitochondrial superoxide levels in organoids (Figure 7D, E).

## Discussion

EGFR mutations are prevalent in tumors of Asian NSCLC adenocarcinoma patients, accounting for approximately 51% 21. While first and second-generation EGFR-TKIs have been the primary choice for initial treatment in patients with sensitive EGFR mutations, the emergence of a secondary T790M mutation in EGFR has led to disease progression in over 50% of cases, limiting the effectiveness of early-generation TKIs 22. Osimertinib, a third-generation EGFR-TKI, has shown promise in targeting T790M-mediated resistance. However, the development of acquired EGFR mutations, particularly C797S, can still lead to resistance 4. Early clinical trials for fourth-generation EGFR-TKIs have demonstrated enhanced potency against T790M/C797S mutants 23. Despite these advancements, EGFR-TKIs alone have not been successful in addressing EGFR-independent resistance mechanisms, such as MET amplification, PIK3CA mutation, BRAF mutation, and others 24. Studies have indicated that patients harboring EGFR mutations in conjunction with other genetic alterations exhibit lower response rates to EGFR-TKIs, as well as shorter progression-free survival (PFS) and overall survival (OS) 25.

Multiple studies on combination of immune checkpoint inhibitors, anti‑angiogenic agents with EGFR-TKIs has progressed to clinical trials. Unfortunately, osimertinib combined with bevacizumab failed to have a prolongation of PFS in advanced NSCLC patients acquiring EGFR T790M mutation 26. Immunotherapy-based combination strategies can bring survival benefit to patients acquiring resistance to EGFR-TKIs. However, severe side effects of the combination limit its clinical application. It is urgent to seek new combined regimens to delay or reverse EGFR-TKIs resistance.

With the rapid advancement of precise targeted therapies, cancer treatment is transitioning from symptomatic approaches to gene therapy. The concept of tumor precision medicine is becoming more prominent in the medical field. While personalized anti-tumor treatments like targeted therapy and immunotherapy have shown promising results, an excessive focus on precision can contribute to the development of more heterogeneous tumors, leading to drug resistance. CSCs, a small cluster of tumor cells, have infinite self-renewal ability and can differentiate into heterogeneous tumor cells 27. Tumor heterogeneity caused by CSCs is a major ongoing challenge in drug resistance, targeting tumor stem cells is a new strategy to reverse drug resistance.

At present, there are many markers for lung cancer stem cells, including functional marker ALDH1 and embryonic stem cell marker SOX2. As a subtype of ALDH1, ALDH1A1 has been found to be significantly upregulated in various cancers such as lung cancer and breast cancer. A Furthermore, ALDH1A1 has been linked to tumor drug resistance, with a study 28 demonstrating that lung cancer cells exhibiting high levels of ALDH1A1 were more prone to developing resistance to gefitinib. Clinical analysis of lung cancer patient samples 29 further supported these findings by revealing elevated expression of ALDH1A1 in tissues that had acquired resistance to gefitinib. SOX2, a member of the Y-related high mobility group (HMG) protein family in SOX region, is a crucial transcription factor for preserving the self-renewal capacity of embryonic stem cells. Additionally, it has been strongly associated with drug resistance and recurrence of lung cancer 30. The results of this study were consistent with previous studies. Following gefitinib intervention in lung cancer models, the mRNA and protein levels of CSCs markers SOX2 and ALDH1A1 were significantly up-regulated.

TRQ is a marketed proprietary Chinese medicine product with antibacterial, anti-inflammatory, and antiviral effects, and is widely used in clinical practice in China. TRQ, a traditional Chinese medicine formula, includes wild honeysuckle flower, baical skullcap root, forsythiae fructus, goral horn, and bear gall powder. The key chemical components of TRQ consist of baicalein, baicalin, rutin, caffeic acid, chenodeoxycholic acid, ursodeoxycholic acid, and chlorogenic acid 31. The main active components of TRQ are baicalein, chlorogenic acid, ursodeoxycholic acid, and chenodeoxycholic acid. Baicalin has demonstrated significant anti-inflammatory effects by modulating the p38 MAPK signaling pathway to reduce the expression levels of IL-1β, IL-6, and TNF-α in lung tissues 32. Furthermore, baicalin has shown inhibition of tumor progression by targeting PI3K/AKT/mTOR, NF-κB and MAPK/ERK signaling pathways 33-35. The combination of baicalin and 5-Fu has been found to inhibit gastric cancer progression by upregulating ROS-mediated iron death 36. Compounds like ursodeoxycholic acid, chenodeoxycholic acid, and chlorogenic acid act on NF-κB, MAPK, JAK-STAT, and other cell signaling pathways, effectively inhibiting the release of inflammatory factors. Chlorogenic acid has been demonstrated to inhibit the progression of colorectal cancer by inducing cell cycle arrest and apoptosis 37. Chenodeoxycholic acid and ursodeoxycholic acid have also exhibited antitumor effects 38-39. TRQ exhibits a broad spectrum of pharmacological activities, including antioxidant, anti-inflammatory, antitumor, and metabolism regulation.

TRQ is commonly used in the treatment of respiratory tract infections, pneumonia, chronic obstructive pulmonary disease, etc. Hu C, *et al.* demonstrated that TRQ treats acute lung injury via lncRNA-SNHG1/HMGB1 40. Research by Jiuling Deng *et al.* showed that TRQ effectively inhibits pulmonary fibrosis by regulating the Sting-mediated endoplasmic reticulum stress signaling pathway 41. At the same time, more and more studies have shown that TRQ has anti-tumor effects. Yang, B. *et al.* pointed out that TRQ injection inhibit the proliferation of leukemia cells and trigger apoptosis 42. There are additional relevant research findings on TRQ's capacity to prevent lung cancer. Ming Ma *et al.* found that TRQ can stimulate lymphocyte proliferation, improve peripheral blood lymphocyte activity in lung cancer patients, and have anti-tumor effects 43. TRQ suppressed the proliferation, invasion, and migration of NSCLC cells by inactivating the HIF1a signaling pathway, which is mediated by exosome circ-WDR78, as revealed by Hong *et al.* Our early study results also demonstrated that combining TRQ with gefitinib reduced AKT and ERK phosphorylation in lung cancer cells, increasing gefitinib's anti-tumor sensitivity 44. This study demonstrated its ability to inhibit the heightened expression of SOX2 and ALDH1A1 induced by gefitinib. Our study also confirmed that combination of TRQ and gefitinib significantly hindered the proliferation of resistant NSCLC models including cell lines, 3D cell spheres, xenograft model and organoids. These results indicate that TRQ has the potential to overcome gefitinib resistance by targeting tumor stemness.

Cancer stem cells mainly rely on glucose metabolism to obtain energy, including glycolysis and oxidative phosphorylation. The redox state of cells is an important process of bioenergy metabolism, and NADH and FAD are important cofactors in the redox process. The sensitive parameter FAD/[NADH+FAD] is commonly used as an indicator of redox metabolism to reflect the redox state of cells 45. An increase in oxidative phosphorylation leads to an increase the ratio, causing cells to shift towards an oxidation state and an increase in ROS levels. Conversely, an increase in glycolysis results in a decrease in this ratio, causing cells to shift towards a reduced state and a decrease in ROS levels 46. Tumor stem cells, in contrast to differentiated tumor cells, rely on glycolysis as ametabolic characteristic to maintain their stemness. Therefore, the increase in redox reactions and ROS levels can promote the differentiation of tumor stem cells 47. It was confirmed that nano-realgar could drive the metabolic reprogramming of CSCs to inhibit the invasion and metastasis of lung cancer 48. Curcumin has been shown to inhibit the stemness characteristics and glucose metabolism of cells, thereby exerting an inhibitory effect on tumors 49. In this study, TRQ combined with gefitinib significantly up-regulated redox ratio of cells and increase ROS level. Depletion of ROS by NAC restored the inhibitory effect of TRQ alone or in combination with gefitinib on stem cell markers SOX2 and ALDH1A1.

STAT3 is a member of the STAT protein family of cytoplasmic transcription factors that can bind to DNA. As the focal point of numerous oncogenic signaling pathways, STAT3 is the most relevant to tumor progression and has been confirmed to be overexpressed in many types of cancer 50. The abnormal and continuous activation of STAT3 in tumor tissue can mediate the signal transduction of various cytokines and growth factors to the nucleus, thereby promoting tumor cell proliferation, inhibiting the tumor cell apoptosis, and inducing chemotherapy resistance 51. Moreover, persistent STAT3 activation is implicated in EGFR-TKIs resistance. Co-administration of selumetinib (AZD6244) and gefitinib can reduce the level of STAT3 activation and increase the sensitivity of NSCLC to gefitinib 52. The dysregulation of intracellular ROS level can induce oxidative stress in cells, consequently impairing the activity of STAT3 pathway and ultimately leading to apoptosis 53. Furthermore, modulation of the ROS/STAT3 signaling pathway has been shown to suppress tumor stemness and overcome drug resistance 54-55. In our study, following the confirmation of ROS's regulation of tumor stemness, we investigated the impact of ROS on STAT3 pathway. The findings revealed that TRQ combined with gefitinib effectively suppressed the activation of STAT3. Scavenging ROS by NAC attenuated the inhibitory effect of TRQ alone or in combination with gefitinib on STAT3 signaling pathway.

In summary, this study demonstrated TRQ synergistically enhanced the antitumor effect of gefitinib on resistant NSCLC models, elevated ROS level, reduced the abnormal activation of STAT3 pathway, thereby inhibiting tumor stemness and overcoming gefitinib resistance. Hence, our findings provided a new combined treatment strategy.

## Figures and Tables

**Table 1:** The clinical features of seven organoids derived from patients with advanced NSCLC

Number	Gender	Age	Pathology	EGFR status	T790M mutation	Genetic aberrations	Lines	Resistance to EGFR-TKIs
**P1**	female	41	LUAD ^(1)^	ex19del	ND ^(2)^	ND	first	yes
**P2**	male	60	LUAD	L858R	yes	*TP53* mutation,	second	yes
						*ALK* mutation		
**P3**	male	77	LUAD	L858R	no	*RB1* mutation,	second	yes
						*TP53* mutation		
**P4**	female	65	LUAD	L858R	yes	*PIK3CA* mutation	fourth	yes
**P5**	female	59	LUAD	L858R	yes	*RB1* mutation,	second	yes
						*PTEN* mutation		
**P6**	female	66	LUAD	L838V,	no	*PIK3CA* mutation,	third	yes
				L861Q		*BRAF* mutation,		
						*TP53* mutation		
**P7**	female	53	LUAD	L858R	ND	ND	fifth	yes

**Note:** (1) LUAD: lung adenocarcinoma; (2) ND: not detection.

## Abbreviations

TRQ: Tanreqing injection
CSCs: cancer stem cell
ROS: reactive oxygen species
STAT3: signal transducer and activator of transcription 3
NSCLC: non-small-cell lung cancer
EGFR-TKIs: Epidermal growth factor receptor tyrosine kinase inhibitors
PC-9-PIK3CA-M: PC-9-PIK3CA-mutation
TCM: traditional Chinese medicine
SOX2: sex determining region Y-box 2
ALDH1A1: aldehyde dehydrogenase family 1 member A1
CCK-8: Cell Counting Kit-8
PBS: phosphate-buffered saline
NAC: N-acetyl-l-cysteine
MPM: multiphoton microscopy
FLI: femtosecond label-free imaging
